# ECG Markers of Acute Melatonin Treatment in a Porcine Model of Acute Myocardial Ischemia

**DOI:** 10.3390/ijms231911800

**Published:** 2022-10-05

**Authors:** Olesya G. Bernikova, Alena S. Tsvetkova, Alexey O. Ovechkin, Marina M. Demidova, Jan E. Azarov, Pyotr G. Platonov

**Affiliations:** 1 Department of Cardiac Physiology, Institute of Physiology, Komi Science Center, Ural Branch, Russian Academy of Sciences, Syktyvkar 167000, Russia; 2 Institute of Medicine, Pitirim Sorokin Syktyvkar State University, Syktyvkar 167001, Russia; 3 Department of Cardiology, Clinical Sciences, Lund University, 22185 Lund, Sweden; 4 V.A. Almazov National Medical Research Center, St. Petersburg 197341, Russia; 5 Arrhythmia Clinic, Skåne University Hospital, 22185 Lund, Sweden

**Keywords:** electrocardiogram, extrasystolic burden, melatonin, myocardial ischemia, ventricular fibrillation

## Abstract

In myocardial ischemia, melatonin confers antiarrhythmic action, but its electrocardiographic expression is unclear. We aimed to evaluate the effects of melatonin treatment on electrocardiogram (ECG) parameters reflecting major arrhythmogenic factors and to test the association of these parameters with ventricular fibrillation (VF) incidence. Myocardial ischemia was induced by 40 min coronary artery occlusion in 25 anesthetized pigs. After induction of ischemia, 12 and 13 animals were given melatonin or placebo, respectively. Twelve-lead ECGs were recorded and durations of QRS, QT, Tpeak-Tend intervals and extrasystolic burden were measured at baseline and during occlusion. During ischemia, VF episodes clustered into early and delayed phases (<10 and >20 min, respectively), and QRS duration was associated with VF incidence. QT interval and extrasystolic burden did not differ between the groups. The Tpeak-Tend interval was progressively prolonged, and the prolongation was less pronounced in the treated animals. QRS duration increased, demonstrating two maxima (5–10 and 25 min, respectively). In the melatonin group, the earlier maximum was blunted, and VF development in this period was prevented. Thus, acute melatonin treatment prevented excessive prolongation of the QRS and Tpeak-Tend intervals in the porcine myocardial infarction model, and QRS duration can be used for the assessment of antiarrhythmic action of melatonin.

## 1. Introduction

Cardiovascular diseases remain the leading cause of death globally. Prediction and prevention of the sudden cardiac death due to ventricular fibrillation (VF) in myocardial infarction remains a challenge, which drives research aimed at identification of novel potential therapeutic methods for treatment of myocardial ischemia/reperfusion injury and protection against ventricular arrhythmias complicating myocardial infarction [1,2,3,4,5,6]. Melatonin is a promising cardioprotective agent with pleiotropic effects [1,2,3,7]. It has been showed to reduce ischemic and reperfusion damage in animal models [8,9,10] and in clinical settings [11,12]. Although melatonin does not influence reentry triggers [13], it has been reported to ameliorate the arrhythmogenic substrate. Specifically, it enhanced ventricular activation [14,15], improved cell coupling [16], reduced adrenergic tone [17], and caused a faster and more complete restoration of action potential duration at reperfusion [14,18]. Collectively, these effects contribute to alleviation of the main arrhythmogenic factors.

Melatonin has shown its potential to reduce coronary artery bypass grafting-related cardiac injury and oxidative stress [19]. Although intravenous and intracoronary melatonin during primary percutaneous coronary intervention in patients with STEMI was not associated with a reduction in infarct size [20], a significant effect appeared in a post hoc analysis for patients who presented early after symptom onset [11].

To date, the antiarrhythmic and electrophysiological effects of melatonin have been assessed only in animal experimental models [13,14,15,18,21,22,23,24,25]. In our previous study, we analyzed the intramyocardial electrograms in the experimental porcine model, and melatonin demonstrated the prevention the early ischemic VF via reduction in conduction delay in the border myocardium [15]. However, melatonin-induced changes in ECG parameters, reflecting the risk of malignant ventricular arrhythmias, were beyond the scope of that work [15] and have not been studied.

The electrocardiogram (ECG) is a widely used tool for dynamic monitoring of the myocardial infarction patients. ECG markers of myocardial ischemia and electrocardiographic manifestations of ventricular arrhythmias are well-known [26]. Several ECG markers, including prolongation of QT, QRS, and Tpeak-Tend intervals, have been shown to be associated with the risk of VF and sudden cardiac death [27,28,29,30]. ECG parameters indicating severity of myocardial injury and arrhythmic propensity may be useful for the monitoring of melatonin effects on the myocardium. A clinically relevant model of acute myocardial infarction in pigs is expected to reveal changes in ECG parameters under the melatonin treatment, which can be subsequently tested in patients with myocardial infarction as a method of assessing control therapy effectiveness.

This study aimed to evaluate the effects of acute melatonin treatment on ECG parameters reflecting major prerequisites of reentrant arrhythmias—dispersion of repolarization (Tpeak-Tend interval), action potential duration (QT interval), conduction velocity (QRS interval), and reentry triggers (ventricular extrasystoles), and to test their association with VF incidence during coronary occlusion in the experimental porcine model.

## 2. Results

### 2.1. ECG Parameters

**Figure 1:**
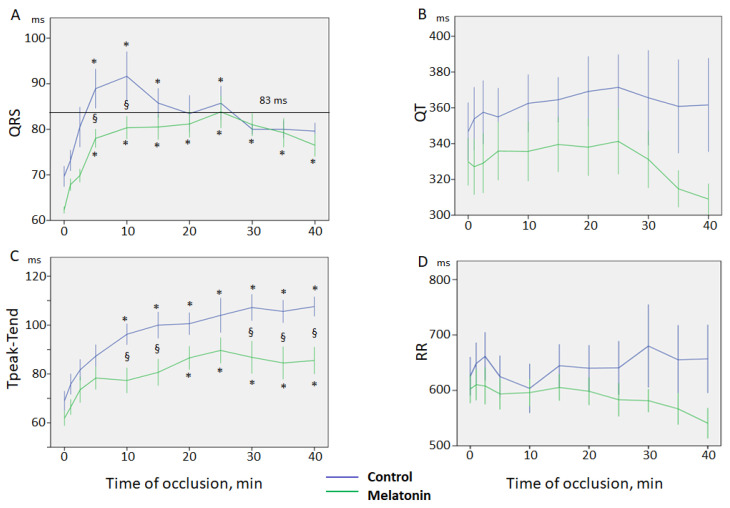
Dynamics of ECG parameters during coronary occlusion in control and melatonin groups (mean ± SEM). (**A**) QRS interval; note two peaks of QRS prolongation at 5–10th and 25th mins. In the melatonin group, the early phase of QRS prolongation is blunted. 83 ms cut-off point of QRS for VF development. Note that QRS duration does not exceed the cut-off point of 83 ms in the melatonin-treated animals; (**B**) QT interval; (**C**) Tpeak-Tend interval; (**D**) RR interval; *-*p* < 0.05 differences in respect to baseline state (0 min); §-*p* < 0.05 differences between control and melatonin groups.

QRS interval was prolonged immediately from the onset of coronary occlusion in both groups. QRS duration demonstrated two maxima at 5–10 min and 25 min. In the animals given melatonin, the early phase of QRS prolongation was reduced. As a result, QRS complex was longer in the control group as compared to the melatonin group at 5–10 min of coronary occlusion and the difference leveled off after 20 min (Figure 1A).

QT interval was 359 ± 5 ms and 331 ± 5 ms in the control and melatonin groups at baseline state, respectively, and did not show significant changes during coronary occlusion (Figure 1B). Tpeak-Tend interval increased progressively during coronary occlusion from 69 ± 4 ms and 62 ± 3 ms, to maxima of 107 ± 5 ms and 89 ± 6 ms, in the control and melatonin groups, respectively. However, the Tpeak-Tend interval was lower in the treated animals and the differences between the groups became significant after 10 min of ischemia (Figure 1C).

There were no differences between the groups in the RR interval duration and it did not change during 40 min of ischemia in the groups of animals (Figure 1D).

### 2.2. Arrhythmias

**Figure 2:**
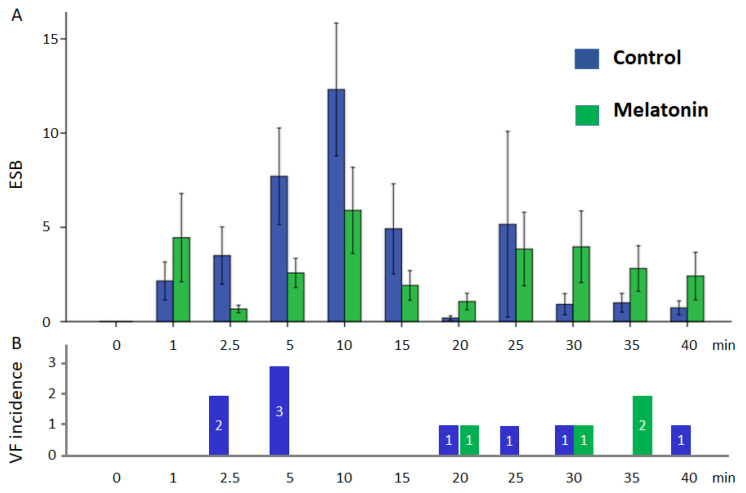
Ventricular arrhythmias in control and melatonin groups during coronary occlusion: (**A**) extrasystolic burden (mean ± SEM); (**B**) number of cases of VF incidence in groups.

**Figure 3:**
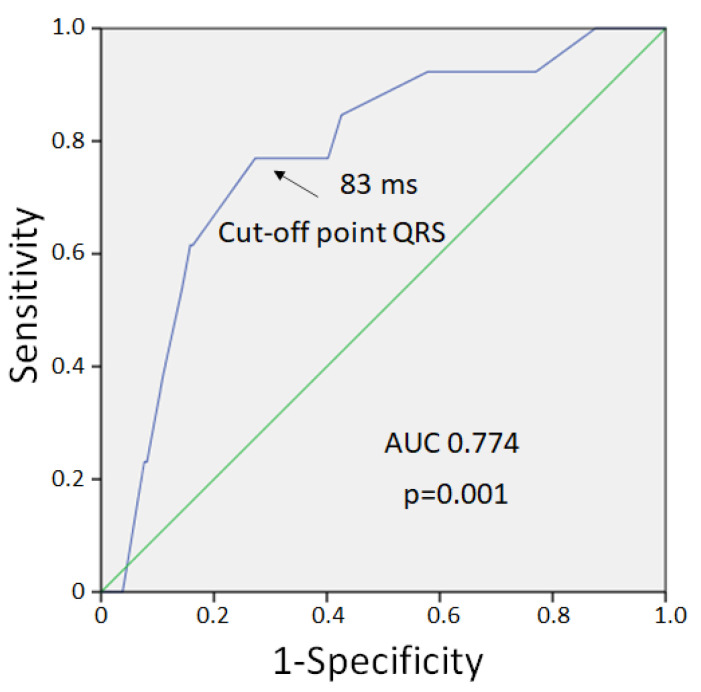
ROC curve analysis of association between QRS duration and VF development.

VF developed in 9 cases out of 13 pigs in the control group and in 4 cases out of 12 pigs in the melatonin group. VFs clustered into two subsets. Early episodes were observed during the first 5 min of occlusion, whereas delayed episodes occurred later (17–40 min) after a silent period (6–16 min). The observed early and delayed VFs corresponded to phase 1A and phase 1B ischemic VF, respectively [31]. The 1A phase VFs were absent in the melatonin-treated animals (0 out of 12 in melatonin group vs. 5 out of 13 in control groups, *p* = 0.016). However, 1B phase VF incidence was similar in the melatonin-treated (4 out of 12 animals) and control (4 out of 8 animals) groups (*p* = 0.456). Extrasystolic burden (ESB) had two maxima at 10 and 25 min (Figure 2) and was significantly associated with VF incidence in the logistic regression analysis (β = 1.092, 95%CI 1.027–1.160, *p* = 0.005). ESB tended to be lower in the melatonin group during the first 10 min of occlusion than in the controls, although did not differ significantly between the groups (Figure 2). When the associations of QRS and Tpeak-Tend intervals with VF incidence were tested, only QRS duration was associated with VF (β = 1.071, 95%CI 1.025–1.118, *p* = 0.002). ROC curve analysis (Figure 3) also demonstrated a significant association between QRS duration and VF incidence (AUC 0.774, *p* = 0.001), and the optimal cut-off for QRS > 83 ms predicted VF development with sensitivity of 0.77 and specificity of 0.73.

## 3. Discussion

The present study demonstrated that melatonin mitigated the ischemia-related prolongation of the QRS complex in the early period of ischemia and that this effect was associated with prevention of the early ischemic VF. Melatonin has been reported to be a promising cardioprotective and antiarrhythmic agent. Numerous experimental studies using either isolated hearts, cardiomyocytes, or an in vivo model showed attenuation of the ischemia/reperfusion-induced myocardial damage and reduction in the incidence of arrhythmias [8,9,14,15,21]. The protective effects of melatonin involve its antioxidant properties [32] as well as receptor-mediated actions [14,15].

Clinical studies showed that melatonin can ameliorate the degree of myocardial ischemic-reperfusion injury related with coronary artery bypass grafting [19,33] and reduce the infarct size after primary percutaneous coronary intervention in patients with STEMI and provided with early melatonin administration [11,20,34]. The difference in melatonin treatment outcomes depended on the time of application during ischemia or reperfusion [11,18,22] and doses of melatonin [4]. In experimental studies, melatonin is applied in doses that are significantly higher than those in clinical trials but still safe for usage in humans [35]. A study of the effect of melatonin on ECG parameters in myocardial infarction is motivated by the necessity to control the therapy effectiveness and estimate the melatonin influence on ECG predictors of life-threatening arrhythmias. In pigs, myocardial infarction develops approximately 7 times faster than in humans [36]. Therefore, the apparently short time of melatonin application (1 min from the onset of occlusion) and phase 1A VF occurrence (≤5 min from the onset of occlusion) in the present porcine model corresponds to a much wider time window regarding humans.

In this study, we evaluated the effects of acute melatonin treatment on ECG parameters reflecting major prerequisites of reentrant arrhythmias—dispersion of repolarization (Tpeak-Tend interval), action potential duration or APD (QT interval), conduction velocity (QRS interval) and reentry triggers (ventricular extrasystoles), and ESB. QRS duration has been demonstrated to be associated with increased all-cause mortality, arrhythmia events, and sudden cardiac death [28]. Although the clinical utility of the Tpeak-Tend interval remains ambiguous [29,37], it has been proven to be a measure of dispersion of ventricular repolarization [38,39]. Prolongation of the QT interval has long been associated with an increased risk of ventricular arrhythmias [27]. The data of meta-analyses conducted in recent decades suggest that QT interval may be an important risk factor for sudden cardiac death [40].

QRS duration significantly prolongs under ischemic conditions, reflecting ischemia-induced ventricular depolarization delay [30,41]. Melatonin treatment was associated with less pronounced QRS prolongation in the present study. In our previous study, we analyzed the intramyocardial electrograms in this porcine model, and melatonin was observed to reduce activation prolongation in the normal and border myocardium when administered at the beginning of an ischemic episode [15]. The shorter myocardial activation times in the melatonin-treated animals correspond to the blunted QRS widening in ECGs found in the present study.

Although exact mechanisms of melatonin action could not be evaluated in this study, several plausible explanations of the observed effects could be provided. We speculate that melatonin may mitigate conduction slowing in the myocardium due to a decrease in ischemia-related depolarization of resting membrane potential [14], which may be due to enhanced IK1 current. This melatonin effect can, in turn, increase the availability of sodium channels and improve propagation. Another probable mechanism of the melatonin effect may be due to preserving connexin properties. Melatonin has been shown to enhance connexin Cx43 expression [42]; however, the realization of this action may be limited by the short period from melatonin infusion to the electrophysiological changes. Moreover, activation of melatonin receptors can prevent connexin lateralization and dephosphorylation, which was reported to be associated with reduction in QRS prolongation in hypokalemic conditions [16].

Effects of melatonin on the repolarization ECG parameters appear to be complex. Melatonin did not modify QT interval duration in our study. The absence of such effects may be due to a weak melatonin influence on APD, if any [14,16,22]. By comparison, melatonin prevented excessive lengthening of Tpeak-Tend interval. The global Tpeak-Tend interval (measured from the earliest Tpeak to the latest Tend throughout all available leads) reflects dispersion of repolarization of the entire ventricular myocardium. Dispersion of repolarization depends not only on APD (which was hardly modified by melatonin) but also on the activation time (which was demonstrated to be affected by melatonin). It is noteworthy that in the study of intramyocardial electrophysiological changes in the pigs [15], we did not find evidence of the acute melatonin effect on dispersion of repolarization, which was probably due to the relatively small region where dispersion of repolarization was measured. This region was limited by the number of intramural leads, which were introduced in the ischemic area and adjacent myocardium, whereas melatonin may also affect remote regions.

Collectively, our data show that melatonin has a prevalent effect on the parameters of depolarization in the ischemic conditions. In the present study, among the tested ECG indices, only QRS interval was associated with VF incidence. This fact was confirmed by studies performed by our group, which showed that myocardial activation parameters strongly correlated with VF incidence in a porcine model of acute coronary occlusion [30,41] and were modified by melatonin [14,15]. In the present study, QRS duration in the melatonin-treated animals who did not develop phase 1A VF did not exceed the cut-off point of 83 ms, which demonstrated the best sensitivity and specificity for association with VF otherwise. Melatonin, however, did not affect the 1B phase VF, which was probably due to the fact that it did not decrease the ESB, which was also associated with VF incidence. These data correspond to our recent findings obtained in a rodent model of ischemia-reperfusion, demonstrating the lack of melatonin effects on the trigger factors [13]. The triggers of reentry arrhythmias occurrence are related to sympathetic activation [43], modification of connexin properties [44], and generation of early or delayed afterdepolarizations [45,46]. It may be speculated that the short-term melatonin treatment in the present experimental model was insufficient to change properties of connexin, APD, or demonstrate sympatholytic effect. Hence, melatonin could not reach the ischemic zone and exclusively affected the perfused myocardium. A preventive melatonin treatment before coronary occlusion may be supposed to lead to electrophysiological effects on ischemic myocardium and have a more pronounced influence on the arrhythmogenic substrate and triggers.

## 4. Materials and Methods

### 4.1. Animal Preparations and Experimental Protocol

In the present investigation, we analyzed ECG data obtained in previous animal experiments described elsewhere [15]. In brief, experiments were performed in 25 Landrace pigs (30–45 kg body weight, both sexes). The study conformed to the ARRIVE guidelines (PLoS Bio 8(6), e1000412, 2010), the Guide for the Care and Use of Laboratory Animals, 8th Edition published by the National Academies Press (USA) 2011, the guidelines from Directive 2010/63/EU of the European Parliament on the protection of animals used for scientific purposes, and was approved by the ethical committee of the Institute of Physiology of the Komi Science Centre, Ural Branch of Russian Academy of Sciences (Russia). The animals were anesthetized with telazol (TELAZOL 100, Zoetis Inc., Parsippany, NJ, USA, 10–15 mg/kg, i.m.) and propofol (Norbrook Laboratories Ltd., Newry, Northern Ireland, UK, 1 mg/kg, i.v.), intubated, and mechanically ventilated.

The thorax was opened by a midsternal incision and the pericardium was cut for further ligature placement around the left anterior descending coronary artery (LAD) just distal to the first diagonal branch origin. Then, the ligature was tightened, and the animals in the melatonin group (*n* = 12) were given melatonin (4 mg/kg, intravenously, Sigma-Aldrich, St. Louis, MO, USA) at the first minute of ischemia. Control animals (*n* = 13) received saline in the amount matching the volume of fluid given to the animals in the intervention group. In the current study, melatonin was administered intravenously after coronary artery occlusion simulating a situation when medication is given after the onset of a heart attack. The chest was reclosed immediately after coronary occlusion. The duration of coronary occlusion was 40 min. The animals were euthanized by potassium chloride infusion under deep anesthesia at the end of the coronary occlusion period or immediately after VF development.

### 4.2. ECG Recordings and Processing

Continuous 12-lead ECG monitoring (KT-07-3/12, INCART, St. Petersburg, Russia) was performed with a sampling rate of 1028 Hz, dynamic range of ±310 mV and 19-bit ADC, which provides an amplitude resolution of 1.18 μV per bit. V1-V6 lead placement corresponded to that in humans.

ECG measurements were performed in each lead at baseline, the 1st, 2.5th, 5th, and 10th, and then every 5 min until the end of 40 min occlusion. The following ECG parameters were determined: RR interval (heart rate), QRS interval, QTp max and QTp min (the longest and shortest intervals between the onset of the QRS complex and the peak of the T-wave in all 12 leads, respectively), and QTe max and QTe min (the longest and shortest intervals between the onset of the QRS complex and the end of the T-wave in all 12 leads). The values from three consecutive beats were averaged.

Analysis of QRS interval was undertaken automatically with subsequent visual control. Two independent investigators (A.S.T. and O.G.B.) performed manual computer-assisted measurements of QTp, QTe, and RR intervals and in case of a difference of >20 ms in each measurement, an agreement was obtained, or a third expert was recruited (A.O.O). Tpeak-Tend interval was taken as the difference between the earliest T-peak (QTp min) and the latest end of the T-wave (QTe max) from all 12 leads at each time point.

Ventricular arrhythmias were evaluated during the ischemia period. For each time-point, starting from 1 min of occlusion, the number of ventricular extrasystoles (ESs) was calculated. VT was considered as three or more consecutive premature ventricular beats. Extrasystolic burden (ESB) was calculated for 1 min (the sum of ES for the time-point period divided by the duration of the period in minutes). In cases of VF and VT occurrence, the ESB was measured for the appropriate time excluding the period of VT and VF. The VF episodes occurring during the first 10 min of ischemia were considered as early or 1A phase VF, while VF cases after 20 min of coronary occlusion were referred to as delayed or 1B phase [31].

### 4.3. Statistical Analysis

Statistical analysis was performed with SPSS package (IBM SPSS Statistics 23, Armonk, North Castle, NY, USA). Data are expressed as mean ± SEM. Parametric tests were used according to the Kolmogorov–Smirnov normality test. Two-way ANOVA with Dunnett post hoc test was used for assessment of ischemia effects on electrophysiological parameters. Comparisons between the control and melatonin groups of animals were undertaken with Student’s t-test. Logistic regression analysis was used to assess the relationships between ECG predictors and VT and VF incidence. The differences were considered significant at *p* < 0.05.

## 5. Conclusions

The present study demonstrated that acute melatonin treatment prevented the excessive QRS and Tpeak-Tend prolongation in a clinically relevant experimental model of myocardial infarction in pigs. From the tested ECG parameters, QRS duration can be used for the assessment of antiarrhythmic action of melatonin.

## Data Availability

The data presented in this study are available on request from the corresponding author. The data are not publicly available due to policy of the Institute of Physiology of the Komi Science Centre, Ural Branch of Russian Academy of Sciences.
